# Hyponatremia and Outcome: Is Severity More Important Than Etiology?

**DOI:** 10.7759/cureus.42808

**Published:** 2023-08-01

**Authors:** Parminder Singh, Saurabh Arora, Diljot Singh, Shivam kalra, Amroz Singh, Utkarsh Arora, Naveen Mittal, Manjeet K Goyal, Simran Kaur, Eva Kalra

**Affiliations:** 1 Endocrinology, Dayanand Medical College and Hospital, Ludhiana, IND; 2 Internal Medicine, Dayanand Medical College and Hospital, Ludhiana, IND; 3 Gastroenterology and Hepatology, Dayanand Medical College and Hospital, Ludhiana, IND

**Keywords:** metabolic abnormalities, metabolic disease, symptomatic hyponatremia, mortality, hypotonic, siadh, hypervolemic, euvolemic, profound hyponatremia

## Abstract

Background and objective: Hyponatremia is the most common electrolyte abnormality encountered in a hospital setting, and the data regarding the contribution of hyponatremia to overall mortality are conflicting. The study objective was to determine patients' clinical profiles and outcomes with hyponatremia.

Methods: This prospective cross-sectional study was conducted at Dayanand Medical College and Hospital, Ludhiana, and included 375 adult patients aged more than 18 years with a confirmed diagnosis of hyponatremia. Patients were subdivided into three groups based on the severity of hyponatremia: mild (130-135 mmol/L), moderate (125-129 mmol/L), and profound (<125 mmol/L).

Results: The most common symptom was confusion (57.3%) followed by deep somnolence (40%) and nausea (36.8%). The most common cause of hyponatremia was diuretics (30.7%), followed by the syndrome of inappropriate antidiuretic hormone secretion (SIADH) (17.8%) and chronic liver disease (CLD) (14.1%). The severity of hyponatremia did not significantly influence the outcome. Patients with CLD and chronic kidney disease (CKD) as the etiology of hyponatremia had significantly worse outcomes compared to other causes of hyponatremia. The most common type was hypovolemic hypotonic followed by euvolemic hypotonic and hypervolemia hypotonic hyponatremia. Nearly half of the total deaths were observed in the hypervolemic hyponatremia group and were significantly higher compared to the other two groups (p=0.001). Correction of hyponatremia (i.e., serum sodium >135 mmol/L) was significantly linked with good outcomes (p=0.003).

Conclusion: Our study showed that the etiology of hyponatremia was a more important prognostic indicator rather than the severity of hyponatremia. Normalization of serum sodium was associated with improved survival.

## Introduction

Hyponatremia (serum sodium concentration: <135 mmol/L) is the most frequent electrolyte abnormality encountered in admitted patients. It can be further classified according to sodium levels (130-135 mmol/L as mild, 125-129 mmol/L as moderate, and <125 mmol/L as profound) or depending upon fluid status (hypovolemic, euvolemic, or hypervolemic) and duration (acute: <48 hours and chronic: >48 hours) [1]. The prevalence of hyponatremia at the time of presentation varies from 5% to 35%, depending on the study population [2-5].

Etiologically, it results from either sodium depletion or excess water in the body, resulting in dilutional hyponatremia, with several hormones playing a role in the pathogenesis [6-7]. Apart from drugs, many disease conditions like chronic liver disease (CLD), chronic kidney disease (CKD), congestive heart failure (CHF), losses from the gastrointestinal tract, neurological disorders resulting in the syndrome of inappropriate antidiuretic hormone secretion (SIADH) or cerebral salt wasting syndrome, malignancy, and endocrine disorders like hypothyroidism and cortisol deficiency can cause hyponatremia [8]. In many cases, more than one factor may contribute to hyponatremia.

In a study conducted by Whelen et al., the severity of hyponatremia was positively correlated with in-hospital mortality risk [9]. A retrospective analysis showed that the mortality rate progressively increased with increasing severity of hyponatremia in patients admitted to intensive care units and was an independent predictor of worse outcomes [10]. These findings could not be replicated in two subsequent studies by Waikar et al. and Chawla et al., where they found increased odds for in-hospital mortality in patients with serum sodium <135 mmol/L, but the trend reversed as the serum sodium fell below 120 mmol/L with a progressive decline in mortality rate [11,12]. Profound hyponatremia, i.e., serum sodium <125 mmol/L, is associated with increased mortality, and there are very few studies assessing the contribution of hyponatremia to overall mortality [10]. The present study was conducted to determine the clinical profile and factors affecting outcomes in patients with hyponatremia.

## Materials and methods

This cross-sectional study was conducted at Dayanand Medical College and Hospital, Ludhiana, India from March 2016 to February 2020, after obtaining approval from the institute's ethical committee. Patients aged >18 years admitted to the Department of Emergency Medicine, medicine wards, intensive care units, and associated medical specialties with laboratory-confirmed hyponatremia were included in the study. Patients with hyponatremia were divided into mild (serum sodium: 130-135 mmol/L), moderate (serum sodium: 125-129 mmol/L), and profound (serum sodium: <125 mmol/L). Patients with profound hyponatremia were further subdivided into three subgroups based on serum sodium levels (subgroup A, 121-125 mmol/L; subgroup B, 116-120 mmol/L; and subgroup C, ≤115 mmol/L). An ion-specific electrode in an electrolyte analyzer estimated serum sodium on the Hitachi Cobas 6000 and Beckman Coulter AU 5800. A detailed clinical history and clinical examination with emphasis on the fluid status of the patients were recorded in the medical notes, along with a detailed drug history. To know the contribution of hyponatremia to mortality, serum sodium levels were monitored regularly during the hospital stay until the time of discharge or death.

The collected data were entered into Microsoft Excel and analyzed using Statistical Package for the Social Sciences (IBM SPSS Statistics for Windows, Version 20.0. Armonk, NY: IBM Corp.). The mean and SD were calculated for quantitative variables, while proportions and percentages were calculated for qualitative variables. A comparison of quantitative variables between the study groups was done using the Student t-test and the ANOVA test. For comparing categorical data, the Chi-square (χ^2^) test was performed. A probability value (p-value) less than 0.05 was considered statistically significant.

## Results

In the present study, 375 consecutive patients of age >18 years admitted with biochemical evidence of hyponatremia were enrolled. Patients were divided into three categories depending on the severity of hyponatremia (mild: 130-135 mmol/L, moderate: 125-129 mmol/L, and profound: <125 mmol/L), and patients with profound hyponatremia were further categorized into three subgroups depending upon serum sodium levels (subgroup A: 121-125 mmol/L, subgroup B: 116-120 mmol/L, and subgroup C: ≤115 mmol/L). A total of 75 patients were included in the mild subgroup and 75 in the moderate categories, whereas 225 patients were enrolled in the profound hyponatremia category (75 in each subgroup A, B, and C).

The age range of 61 to 80 years comprised the majority of patients in all three groups (n=196, 52.2%), with the 41 to 60 age range coming in second (n=130, 34.7%). The mean age in the mild, moderate, and profound categories was 61.2 years, 58.3 years, and 62.4 years, respectively, with no statistically significant difference among the three groups. Out of 375 patients, 226 patients (60.3%) were males and 149 patients (39.7%) were females, with a male preponderance seen in all three categories, i.e., 60%, 64%, and 59.1% in the mild, moderate, and profound categories, respectively. The baseline characteristics of all patients are shown in Table 1

**Table 1:** Baseline characteristics of patients in all three groups

Parameter	Mild hyponatremia (Na=130-135 mmol/L)	Moderate hyponatremia (Na=125-129 mmol/L)	Profound hyponatremia (Na<125 mmol/L)
Number of patients	75	75	225
Age (years, mean±SD)	61.2±16.3	58.3±15.3	62.4±13.7
Male to female ratio	1.5	1.7	1.4
Diabetes mellitus, n (%)	24(32)	22(29.3)	89(39.5)
Hypertension, n (%)	34(45.3)	36(48)	112(49.7)
Bronchial asthma, n (%)	8(10.6)	4(5.3)	5(2.2)
Hypovolemic, n (%)	25(33.3)	23(30.6)	90(4)
Euvolemic, n (%)	23(30.6)	18(24)	68(30.2)
Hypervolemic, n (%)	22(29.3)	30(40)	54(24)
Serum sodium (mmol/L, mean±SD)	133.44±1.78	128.01±2.92	117.63±3.88
Length of hospital stay (days, mean±SD)	11.46±7.16	12.24±6.88	12.76±7.21

A total of 75.7% (n=284) patients were found to be symptomatic, with a maximum of 88.8% (n=200) in the profound hyponatremia group, 58.7% (n=44) in the moderate hyponatremia group, and 53.3% (n=40) in the mild hyponatremia group. In all three subgroups of patients with profound hyponatremia, the majority had both moderately severe and severe symptoms, which were classified based on clinical practice guidelines on diagnosis and treatment of hyponatremia developed by the European Society of Intensive Care Medicine (ESICM), the European Society of Endocrinology (ESE), and the European Renal Association-European Dialysis and Transplant Association (ERA-EDTA) [1]. The most common symptom was confusion (n=215, 57.3%), followed by deep somnolence (n=150, 40%). Nausea and vomiting were the other common symptoms present in 36.8% (n=138) and 27.2% (n=102) of patients, respectively. Headache, seizures, and coma were present in 10.6% (n=40), 6.6% (n=25), and 4% (n=15) of the patients, respectively. The most commonly associated co-morbidity was hypertension, which was seen in 182 patients (48.5%), followed by diabetes mellitus in 135 patients (36%).

Diuretic use (30.7%), SIADH (17.8%), and CLD (14.1%) were the three most prevalent causes of hyponatremia in our study. The most common cause in all three groups separately was diuretic use only (mild: 29.3%, moderate: 28%, and profound: 32%). Twenty-five patients (6.6%) out of 375 had two or more precipitating factors as the cause of hyponatremia. The mean length of hospital stay for patients in the mild, moderate, and profound categories was 11.46, 12.24, and 12.76 days, respectively. The mean length of hospital stay increased with the severity of hyponatremia, but it was statistically insignificant.

Out of 375 patients with hyponatremia, the most common type of hyponatremia was the hypovolemic hypotonic type, seen in 138 patients (36.8%), of which diuretics alone was the most common precipitating factor in 86 patients (62.3%). 109 patients (29%) had euvolemic hypotonic hyponatremia, with SIADH (61.4%) being the most common cause, followed by low solute intake. Hypervolemic hypotonic hyponatremia was encountered in 106 patients (28.2%), with CLD accounting for 47.1% of cases in this group, followed by CKD (37.7%). Other causes of hypervolemic hyponatremia in our study were heart failure, hypoproteinemia, and acute kidney injury. Twenty-two patients could not be categorized due to an undetermined volume status with mixed etiology as shown in Table 2.

**Table 2:** Etiological analysis of patients in all three groups CLD, chronic liver disease; CKD, chronic kidney disease; CHF, chronic heart failure

Parameter	Mild hyponatremia (Na=130-135 mmol/L)	Moderate hyponatremia (Na=125-129 mmol/L)	Profound hyponatremia (Na<125 mmol/L)
Diuretic use, n (%)	22(29.3)	21(28)	72(32)
Thiazides, n (%)	17(22.67)	22(29.34)	37(49.34)
Loop diuretics, n (%)	9(12)	12(16)	16(21.3)
Aldosterone antagonists, n (%)	0	0	2(2.6)
SIADH, n (%)	13(17.3)	11(14.6)	43(19.1)
Low solute intake, n (%)	10 (13.3)	5(6.6)	20(8.8)
CLD, n (%)	10(13.3)	18(24)	25(11.1)
CKD, n (%)	9(12)	10(13.3)	21(9.3)
CCF, n (%)	3 (4)	4(5.3)	14(6.2)
Hypocortisolism, n (%)	2 (2.6)	1(1.3)	10(4.4)
Hypothyroidism, n (%)	1(1.3)	1(1.3)	4(1.7)
Mixed etiology, n (%)	5(6.6)	4(5.3)	16(7.1)

Out of 375 patients, 318 (84.8%) were discharged, and 57 (15.2%) died. Statistical analysis revealed that age, sex, and serum sodium level at presentation did not significantly affect the outcome of patients. Out of 57 patients who died, 10 (13.3%) were from the mild hyponatremia group, 11 (14.6%) from the moderate hyponatremia group, and 36 (16%) from the profound hyponatremia group, indicating a trend toward increased mortality but not statistically significant (p=0.52). Furthermore, even going deep into the profound hyponatremia group, mortality in all three subgroups did not differ significantly (p=0.153). Out of the 318 patients who were discharged, 64.8% (206 patients) had almost corrected hyponatremia (serum sodium: >135 mmol/L), and 43.8% (25 patients) of patients who died had corrected hyponatremia on the day of demise. Failure to correct hyponatremia (last sodium: <135 mmol/L) was significantly associated with poor outcomes (p=0.003).

Patients with CLD and CKD as the etiology of hyponatremia had poor outcomes compared to patients with other causes of hyponatremia. Out of 53 patients with CLD, 17 (32%) died. The mortality rate was significantly higher in patients with hyponatremia due to underlying CLD (p=0.004). Similarly, patients with hyponatremia caused by CKD had significantly higher mortality (27.5%, p= 0.025), whereas mortality was lower in the other groups (SIADH: 11.9%, diuretics: 6.9%, and heart failure: 4.8%). Out of the 57 patients who died, 29 (50.9%) were in the hypotonic hypervolemic hyponatremia group, followed by 16 (28.1%) patients in the hypotonic hypovolemic group, and 12 (21%) patients in the hypotonic normovolemic group. Thus, about half of the total mortality was in the hypervolemic hyponatremia group and was significantly higher compared to the other two groups (p=0.001). Univariate analysis of variables associated with the outcome of patients with hyponatremia is shown in Table 3.

**Table 3:** Analysis of possible risk factors associated with mortality CLD, chronic liver disease; CKD, chronic kidney disease; CHF, chronic heart failure

Risk factor	Odds ratio	95% confidence interval	p-value
CLD	3.54	1.71-6.47	0.004
CKD	2.38	1.11-5.09	0.025
CHF	2.03	1.23-4.67	0.023
Hypervolemic hyponatremia	3.24	1.81-5.78	0.001
Failure of correction of hyponatremia (last serum sodium: <135 mmol/L)	2.35	1.32-4.16	0.003
The severity of hyponatremia (serum sodium: <125 mmol/L)	1.17	0.65-2.09	0.52

## Discussion

The present study enrolled patients with hyponatremia and examined the association between the severity of hyponatremia and the outcome of the patients. Additionally, the study also determined the etiology, clinical features, and contribution of hyponatremia toward mortality. Our study demonstrated that the severity of hyponatremia at the time of admission was not significantly associated with increased mortality. Rather, correction of hyponatremia at the time of discharge was shown to improve the outcome. The etiology of hyponatremia emerged as a key parameter determining the outcome of hospitalized patients.

The mean age of patients in the mild, moderate, and profound categories was 61.2 years, 58.3 years, and 62.4 years, respectively, with no statistically significant difference among the three groups. Our results matched those of a prospective observational study conducted in a tertiary care hospital by Pandey et al. in which hyponatremia was found to be more prevalent among elderly patients than in younger patients [13]. In a study conducted by Tierney et al., the mean age of presentation was 61 years, whereas in a cohort study of 4123 older patients by Terzian et al., the mean age of presentation was 77 years [14,15]. In the current study, 226 patients (60.3%) were males and 149 patients (39.7%) were females, with a uniform male preponderance seen in all three groups. This is in concordance with a study conducted by Chatterjee et al. in a tertiary care hospital in Eastern India in which there were 126 (62.69%) male patients and 75 (37.31%) female patients [16].

The symptoms of hyponatremia can range from nausea and malaise with a mild reduction in sodium levels to life-threatening features like seizures and coma [10]. Out of 375 patients, a total of 284 (75.7%) were found to be symptomatic, with a gradual increase in the symptomatology with increasing severity of profound hyponatremia (53.3% in the mild hyponatremia group, 58.7% in the moderate hyponatremia group, and 88.8% in the profound hyponatremia group). The majority of patients in the profound hyponatremia group had both moderately severe and severe symptoms. The most frequent symptom was confusion (57.3%), then deep sleep (40%) and nausea (36.8%). Headache, seizures, and coma were present in 10.66%, 7%, and 5% of the patients, respectively. The frequency of headaches observed by Agrawal et al. was high (40%) compared to our study, whereas confusion was reported in a lesser number of patients (41%) [17]. Rao et al. reported headaches and seizures in 8% and 5%, respectively [18].

Diuretic use (30.7%) was the most common causative factor implicated in the development of hyponatremia in the current study. Other common causes of hyponatremia included SIADH (17.8%), CLD (14.1%), and CKD (10.7%). The most common cause separately in all three groups was diuretics (mild: 29.3%, moderate: 28%, and profound: 32%). This finding is in line with the results of a study conducted by Maqbool et al. in which they found diuretic use to be the most common cause of hyponatremia (34%), followed by SIADH (29%), gastrointestinal losses (10%), and CKD (8%) [19]. Similar findings by Clayton et al. indicate that the use of diuretics, particularly thiazide diuretics, is one of the most frequent causes of hyponatremia [20]. This highlights the importance of a dedicated drug history in the evaluation and management of patients with hyponatremia. Studies conducted by Babaliche et al. and Pillai et al. reported SIADH as a predominant cause of hyponatremia in patients admitted to the intensive care unit [21,22].

In our study, hypovolemic hypotonic hyponatremia was the most prevalent type (36.8%), then euvolemic hypotonic hyponatremia (29%), and finally hypervolemic hypotonic hyponatremia (28%). These findings are in agreement with the results observed in the study by Cumming et al. in which they found the majority of patients had hypovolemic hyponatremia (69.7%), and the most common potentially causative factor in their study was thiazide diuretics (76%) [23]. Euvolemic hyponatremia followed this in 27.3% of patients, whereas hypervolemic hyponatremia only affected 3% of patients. Another study conducted by Padhi et al. showed that euvolemic hyponatremia was the most common cause of hyponatremia in critically ill patients [24]. A direct comparison of different studies is not possible owing to the heterogeneity of the populations involved in various studies. The most common type of hyponatremia overall is euvolemic hyponatremia, which represents 60% of all patients with hyponatremia [25].

The relationship between the severity of hyponatremia and mortality risk has always been a matter of debate, with various studies reporting conflicting results. Our study showed a tendency toward increased mortality risk when analyzed for the severity of hyponatremia (i.e., out of 57 patients who died, 10 (13.3%) were from the mild group, 11 (14.6%) from the moderate group, and 36 (16%) were from the profound group), but it was not statistically significant. Even going deep into the subgroup analysis of patients with profound hyponatremia revealed that the severity of hyponatremia was not significantly associated with mortality. Our findings reflect that the severity of profound hyponatremia did not have any correlation with the outcome of patients. The results of our study are similar to those of Gill et al. and Stern RH, suggesting that the severity of hyponatremia is not the predominant factor linked to mortality in these patients [26,27]. Contrary to our observation, Erasmus and Matsha found an increased mortality rate with increasing severity of hyponatremia [28].

Our breakdown of mortality data on the basis of etiology suggests that patients with CLD and CKD as etiology of hyponatremia had significantly worse outcomes compared to other causes of hyponatremia. The findings of the present study suggest that the etiology of hyponatremia is a more important prognostic factor than absolute sodium levels. Our observation is in agreement with the findings of Clayton et al., highlighting the fact that the outcome is governed principally by underlying disease-causing hyponatremia [29]. Nearly half of the total patients who died belonged to the hypervolemic hyponatremia group, and this was significantly higher compared to the normovolemic and hypovolemic groups. This is possible because of the underlying organ dysfunction (i.e., hepatic, renal, and cardiac) in this group, and therapeutic options in these patients are generally limited.

Whether the correction of hyponatremia is associated with improved survival has not been clearly ascertained so far. Our study showed that improvement in hyponatremia (serum sodium: >135 mmol/L) was significantly associated with better outcomes (p=0.003). A recent meta-analysis including 13,816 patients with hyponatremia showed that correction of hyponatremia significantly reduced mortality in hospitalized patients (p=0.002), and the results were even more remarkable after performing a sensitivity analysis, which included only those studies (n=8) reporting a cutoff for serum sodium improvement of >130 mmol/L (p<0.001) [30]. Another study in patients with congestive cardiac failure reported increased mortality in the absence of hyponatremia correction, and this appeared to be independent of cardiac dysfunction severity. In addition, some shortcomings of our study must be recognized. The current study design did not allow for the compilation of complete data on the rate of correction of hyponatremia or chronicity of hyponatremia, and we recognize that both of these factors can act as potential confounders in the assessment of outcome.

## Conclusions

Overall, this study demonstrates that the outcome of profound hyponatremia is governed primarily by the underlying disease, and not by the severity of hyponatremia. The hypervolemic hyponatremia group was associated with significantly increased mortality compared to the euvolemic and hypovolemic groups. Additionally, correction of hyponatremia is associated with decreased mortality, although the cause-effect relationship could not be determined.
